# Preliminary evaluation of deep learning for first-line diagnostic prediction of tumor mutational status

**DOI:** 10.1038/s41598-023-34016-y

**Published:** 2023-04-28

**Authors:** Louis-Oscar Morel, Valentin Derangère, Laurent Arnould, Sylvain Ladoire, Nathan Vinçon

**Affiliations:** 1 Ummon HealthTech, Dijon, France; 2 Centre Georges François Leclerc, Dijon, France

**Keywords:** Diagnostic markers, Tumour biomarkers

## Abstract

The detection of tumour gene mutations by DNA or RNA sequencing is crucial for the prescription of effective targeted therapies. Recent developments showed promising results for tumoral mutational status prediction using new deep learning based methods on histopathological images. However, it is still unknown whether these methods can be useful aside from sequencing methods for efficient population diagnosis. In this retrospective study, we use a standard prediction pipeline based on a convolutional neural network for the detection of cancer driver genomic alterations in The Cancer Genome Atlas (TCGA) breast (BRCA, n = 719), lung (LUAD, n = 541) and colon (COAD, n = 459) cancer datasets. We propose 3 diagnostic strategies using deep learning methods as first-line diagnostic tools. Focusing on cancer driver genes such as KRAS, EGFR or TP53, we show that these methods help reduce DNA sequencing by up to 49.9% with a high sensitivity (95%). In a context of limited resources, these methods increase sensitivity up to 69.8% at a 30% capacity of DNA sequencing tests, up to 85.1% at a 50% capacity, and up to 91.8% at a 70% capacity. These methods can also be used to prioritize patients with a positive predictive value up to 90.6% in the 10% patient most at risk of being mutated. Limitations of this study include the lack of external validation on non-TCGA data, dependence on prevalence of mutations in datasets, and use of a standard DL method on a limited dataset. Future studies using state-of-the-art methods and larger datasets are needed for better evaluation and clinical implementation.

## Introduction

Targeted cancer therapies are specialized and efficient therapies that have revolutionized the treatment of cancer in the last few years^1,2^. The higher specialization of targeted cancer therapies requires to know more and more information about the patient. Getting personalized information requires using more specialized diagnostic tests^3^. As an example, the presence or the absence of genomic mutations can be associated with a response to a targeted cancer therapy like Wee1^4^ inhibitors are treatments that are efficient only on cancers for which *TP53* is mutated. Detection of somatic mutation is routinely made by DNA-sequencing. However, these tests face a three fold limitation: they have a long waiting period, require a large amount of tissue and are expensive. Therefore, there is a growing need to identify new biomarkers, associated screening and diagnostic strategies to improve efficiency of diagnostic workflows in medical oncology.

More recently, deep learning methods have been used for many image analysis tasks in digital pathology such as tumour detection^5^, tumour subtyping^6^, quantification of cell numbers^7^ and classification of cell types^8^, RNA-seq^9^ and have shown promising results for the prediction of the mutational status from digitized tissue stained with hematoxylin and eosin as whole slide images (WSI)^10–15^. The seminal work of Coudray et al.^10^ showed that key mutations of lung cancer could be identified from histopathology slides. Many other studies have followed and have shown similar results in brain^16,17^, bladder^18,19^, colorectal^14,20,21^, breast^22,23^, gastric^24,25^, liver^26,27^ and also in pan-cancer studies^28^, demonstrating the presence of a link between histomorphology and genetic features. These studies mostly report AUC, a metric that is efficient to compare different approaches but that is not relevant to evaluate the benefits of the method in clinical routine.

These WSI are already made routinely in the diagnostic workflow and deep learning methods are cost-effective, always feasible and highly scalable. Therefore, a deep learning based solution assessing the tumoral mutational status of a patient directly onto the WSI appears as a potentially valid diagnostic strategy. Here, we evaluate the benefits of using a standard deep learning pipeline for mutational status prediction on WSI in the diagnostic strategy for patients with breast, lung and colorectal cancer. We simulate three diagnostic strategies using the deep learning pipeline as a first-line diagnostic tool in a clinical context before using DNA sequencing^29^.

The first strategy “Save-all” considers the number of diagnostic tests that can be avoided while preserving a high sensitivity. The second strategy “Fixed-Capacity” considers, in the case of a limited number of diagnostic tests available, the proportion of the positively mutated patients found (sensitivity) during DNA sequencing. In other words, it optimizes the number of patients that will later benefit the associated targeted therapy for a limited DNA testing capacity. The last strategy “Prioritization” considers the number of mutated patients found in a small part of the patient population for short-tracking. The rationale behind this strategy is that the earlier the patient has access to the best therapy, the higher might be its chance of remission^30,31^.

We finally show the relevance of our deep learning algorithm for each strategy in these realistic screening scenarii by showing its efficiency for each gene that both has a predictable mutational status and is clinically relevant and demonstrate the efficiency of the “fixed-capacity” strategy to reduce population inequalities.

## Material and methods

### Study design

This study is retrospective and uses anonymized scanned WSIs retrieved from the TCGA project through the Genomic Data Commons Portal (https://portal.gdc.cancer.gov/). We applied our method to the following tumor types: breast (BRCA), lung (LUAD), colon (COAD). The clinical data is registered with dbGaP study accession number: phs000178.v11.p8. For patient information, please refer to the original TCGA publications^32–34^.

All experiments were conducted in accordance with the Declaration of Helsinki and the International Ethical Guidelines for Biomedical Research Involving Human Subjects. Ethics oversight of the TCGA study is described at https://www.cancer.gov/about-nci/organization/ccg/research/structural-genomics/tcga/history/policies. Informed consent was obtained by all participants in the TCGA. We followed the Standards for Reporting Diagnostic Accuracy Studies (STARD) guidelines to ensure comprehensive and transparent reporting of our study methods and results^35^.

### Datasets

All data, including histological images and information about the participants from the TCGA database are available at https://portal.gdc.cancer.gov/. Genetic data for patients in the TCGA cohorts are available at https://portal.gdc.cancer.gov/. The corresponding authors of this study are not involved in data sharing decisions of the TCGA database. The TCGA-BRCA dataset included 719 slides from 34 different centers, the TCGA-LUAD dataset included 541 slides from 33 different centers and the TCGA-COAD dataset included 459 slides from 24 different centers. All available data were used for subsequent analyses.

### Molecular labels

Molecular labels were determined from the masked somatic mutations maf file of somatic mutation using the MuTect2^36^ algorithm corresponding to the dataset. A gene having a mutation with an IMPACT value “HIGH” or “MODERATE” categorized by VEP software^37^ was considered positive while other IMPACT values or no mutation were considered negative. A positive label is encoded as 1 and a negative label is encoded as 0. Protocol for DNA sequencing can be found here : https://www.cancer.gov/ccg/research/genome-characterization-pipeline. No label was missing.

### Pipeline

All analyses were performed using Python 3.8.

### Image preprocessing

Aperio SVS files of formalin-fixed paraffin-embedded (FFPE) diagnostic slides (labeled by a “DX” in their name) from the 3 datasets were first selected. We did not exclude any diagnostic slide from the analysis. We then extracted the foreground using an in-house trained U-net, and tiled the images in non-overlapping patches of 600 × 600 pixels at a 5 × resolution, thus leading to 331,263 patches for TCGA-BRCA dataset, 302,544 patches for TCGA-LUAD dataset and 178,224 patches for TCGA-COAD dataset. These patches are associated with the label 1 if the gene is mutated, 0 if the gene is non mutated according to the “Molecular labels” section above. We then used an EfficientNetB7^38^ neural network truncated at its last layer on which we added a global average pooling layer in order to output an embedding vector of size 2560.

### Gene prediction from histopathology

For each of the 3 datasets, we selected all genes having at least one mutation in more than 10% of the slides in the dataset and we divided the dataset in a train set (70%) and a test set (30%) without any overlap between cancer site between train set and test set following Howard et al*.* recommendations for train and test splitting^39^ and stratified for positive and negative slides. Tissue source site was provided by the barcode of the TCGA slides^40^. For each of these genes, we created an ensemble of 3 multi-layer perceptrons (MLP) with 2 intermediate layers of 64 and 16 neurons with ReLU activation function and one final layer with 1 output with a sigmoid activation function. These 3 MLP were trained for 5 epochs on the train set using the Adam optimizer with a learning rate of 1e-4 and the binary cross entropy loss, then applied on the test set and their prediction values were averaged between the 3 MLP for each tile. The slide-level predictions were calculated using the 99th percentile of its tile prediction values. Each gene prediction was run 5 times with random initialization and AUC were tested against the theoretical random value of 0.5 with a one-sided Student t-test, assuming that the variability followed a normal distribution. Corrections for multiple hypotheses were made using the Benjamini-Hochberg^41^ correction procedure with a false discovery rate of 0.05.

### Cancer driver genes metrics estimation

We used the gene set described in the “Gene prediction from histopathology” section and restricted the diagnostic strategies analysis to cancer driver genes. We defined the cancer driver genes as being annotated as “Oncogene” or “Tumor suppressor” in the OncoKB database^42^. We divided the dataset in 5 folds without any overlap between cancer sites between folds for cross-validation following Howard et al*.* recommendations for train and test splitting^39^. Tissue source site was provided by the barcode of the TCGA slides^40^. For each of these genes, we created an ensemble of 3 multi-layer perceptrons (MLP) with 2 intermediate layers of 64 and 16 neurons with ReLU activation function and one final layer with 1 output with a sigmoid activation function. These 3 MLP were trained for 5 epochs on the train set using the Adam optimizer with a learning rate of 1e-4 and the binary cross entropy loss, then applied on the test set and their prediction values were averaged between the 3 MLP for each tile. The slide-level predictions were calculated using the 99th percentile of its tile prediction values. Mean and standard deviation of the mean were calculated for each metric of interest described in the next section (Diagnostic strategies).

### Implementation and hardware

Experiments were run with a NVIDIA RTX A4000 graphic card and the following libraries : TensorFlow v2.8.0-rc0, keras v2.8.0, CUDA 11.5.

### Diagnostic strategies

#### Save-all

This strategy reduces the overall diagnostic cost of a population.

In the “Save-all” strategy, the DL-based test is used as a first-line diagnostic tool. Access to gold standard sequencing tests is conditioned by the positivity of the DL-based test. Reduction of the cost is gained because fewer patients are being sequenced. This strategy is weighted by a loss representing the false negative rate of the deep learning pipeline (patient negative during screening but normally positive during sequencing).

In the “Save-all” strategy, we try to eliminate non-mutated patients while having the least false-negative to avoid unnecessary diagnostic tests. More specifically, we are interested in the number of diagnostic tests that can be avoided while preserving a high sensitivity. The number of allowed false negatives is defined by a sensitivity threshold of 95%. (Fig. 2A). Uncertainties (shown after the ± symbol) are standard deviations of the mean.

We have then:

$$\mathop {max}\limits_{Se\left( X \right) \ge T} N\left( {DL} \right)$$

with $T = 95\%$, $N\left( {DL} \right)$ the proportion of negative DL-based tests, $Se\left( X \right)$ the sensitivity of the full pipeline (DL + sequencing).

#### Fixed capacity

This strategy optimizes the allocation of a fixed budget of gold standard sequencing-based tests.

In the “Fixed-Capacity” strategy, the DL-based test is used as a first-line diagnostic tool, then access to gold standard sequencing tests is conditioned by the positivity of the DL-based test as in the “Save-all” strategy. The gain is an increase in the number of patients that can benefit the treatment that are effectively found.

In the “Fixed-Capacity” strategy, we optimize the number of mutated patients found in the case of a limited number of sequencing tests (fixed budget). In this configuration, the goal is to optimize the number of patients that will finally benefit from the associated targeted therapy. More specifically, we are interested in the full diagnostic pipeline sensitivity while the number of positive DL-based tests is restricted (i.e. the number of patients that will have access to the sequencing-based test). The proportion of available sequencing-based tests is defined to 30%, 50% or 70% (Fig. 2BC). Uncertainties (shown after the ± symbol) are standard deviations of the mean.

We have then :

$$\mathop {max}\limits_{{P\left( {DL} \right) \le T}} Se\left( X \right)$$

with $T = 30\% , 50\% or 70\%$, $P\left( {DL} \right)$ the proportion of positive DL-based tests, $Se\left( X \right)$ the sensitivity of the full pipeline (DL + sequencing).

#### Prioritize

This strategy increases the overall clinical value by treating patients with high-risk of mutation earlier.

In the “Prioritize” strategy, the DL-based test is used as a first-line diagnostic tool, then there are two types of access to the gold standard sequencing tests : the early access (priority line) and the normal access. The rationale behind the “Prioritize” strategy is that in a context of cancer, time is against the patient and days can significantly change the prognosis^30,31^. Therefore, the gain is an increase in the clinical value for the patient that will have access to the best therapy earlier and will have a better prognosis.

In the “Prioritize” strategy we select a small proportion of patients that are highly likely to be positive to prioritize them for the sequencing diagnostic test. More specifically, we are interested in the positive predictive value (PPV) while the number of positive DL-based tests is restricted (i.e. the number of patients that will have access to an early sequencing-based test) to a cutoff. We defined the cutoff at 5% and 10% because it is high enough to have an influence at the population level and low enough to be realistic for a short-track. Uncertainties (shown after the ± symbol) are standard deviations of the mean.

We have then:

$$PPV\left( {DL} \right) where\mathop {max}\limits_{{P\left( {DL} \right) \le T}} P\left( {DL} \right)$$

with $T = 5\% or 10\%$, $P\left( {DL} \right)$ the proportion of positive DL-based tests, $PPV\left( {DL} \right)$ the positive predictive value of the DL-based test.

We calculated the relative risk (RR) as the PPV over the prevalence. The uncertainty measure (shown after the ± symbol) is the standard deviation of the PPV over the prevalence.

## Results

### A significant proportion of mutations are detectable with deep learning methods on whole-slide imaging

**Figure 1:**
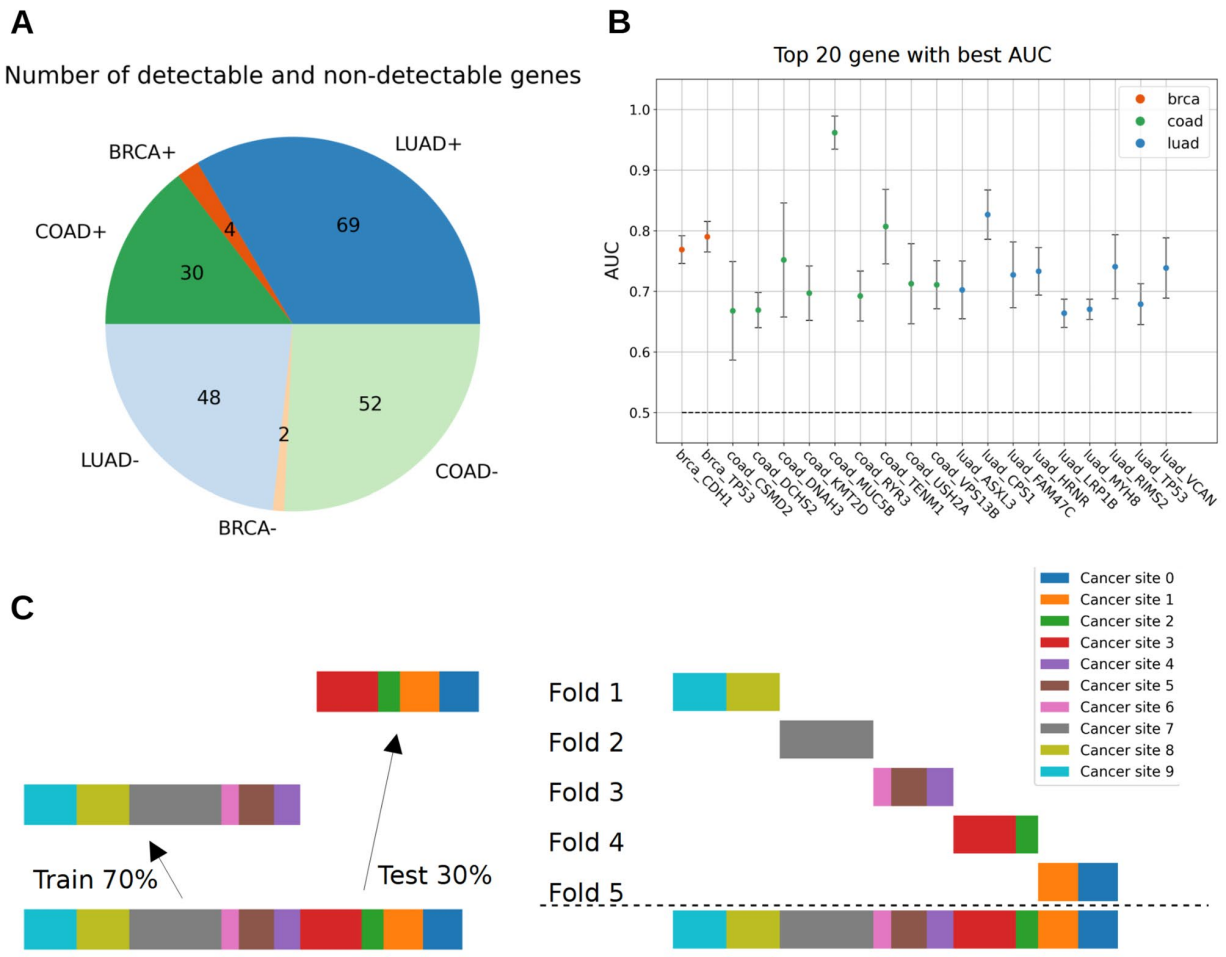
Description of predictable genes in TCGA-LUAD, TCGA-COAD and TCGA-BRCA. (**A**) Pie of the number of genes according to their dataset and their predictability (see Material and Methods). Predictable genes are shown in dark color with “+”, other genes are shown in light color with “-”. Statistical significance was determined by a Student t test corrected with Benjamini–Hochberg for multiple hypothesis testing (see Methods for further details). The displayed number is the number of genes within each category of the pie. (**B**) Scatter plot of the AUC of predictable genes, only the 20 genes with top AUC were selected for clarity. Error bars are the standard deviation of the AUC over 5 repetitions with random initialization. The dashed bar at 0.5 corresponds to the theoretical AUC of a random classifier. Dataset of origin is shown in color with the same color code as in the figure slot A. (**C**) Description of the train-test split for gene prediction on the left and the fivefold split for metric estimation on the right. The split ensures that there is no overlap of cancer sites between train and test in order to avoid biases due to batch effect. The 10 cancer sites are shown to illustrate the general idea but do not represent the true cancer sites.

We first set up a deep learning pipeline predicting solid tumor gene mutations from WSI using an EfficientNetB7^38^ pre-trained on ImageNet dataset. In this study, 3 datasets from GDC Portal were analyzed, a lung adenocarcinomas dataset, a colorectal cancer dataset and a breast carcinoma and adenocarcinoma dataset, respectively TCGA-LUAD with 522 patients (541 slides), TCGA-COAD with 459 patients (459 slides), TCGA-BRCA with 687 patients (719 slides). Tables 1 and 2 provide a comprehensive overview of the patient population, including their demographic and clinical characteristics, as well as disease features. In order to validate the ability of our deep learning pipeline to predict gene mutations, we systematically trained the deep learning pipeline on every mutation having a prevalence greater than 10% in the dataset to focus on mutations that can show statistical significance, these correspond to 205 tested genes. For each gene we split the dataset in a train set (70%) and a test set (30%) from distinct cancer sites (see Methods). We found that 50% of the gene had a statistically significant predictable mutational status with our baseline DL pipeline (one-sided Student t-test corrected using Benjamini–Hochberg procedure with a FDR of 0.05, Fig. 1). However, given the low number of samples, especially for mutation with low prevalence, one can expect that more of these effectively have a predictable mutational status. Therefore, this validates the ability of our model to predict gene mutations from WSI.

**Table 1:** Population characteristics.

	TCGA-BRCA	TCGA-LUAD	TCGA-COAD
Number of patient	687	522	459
Ethnicity			
Hispanic or latino	26 (3.8%)	7 (1.3%)	4 (0.9%)
Not hispanic or latino	560 (81.5%)	389 (74.5%)	271 (59.0%)
Not reported	101 (14.7%)	126 (24.1%)	184 (40.1%)
Gender			
Female	681 (99.1%)	280 (53.6%)	216 (47.1%)
Male	6 (0.9%)	242 (46.4%)	243 (52.9%)
Race			
American indian or alaska native	1 (0.1%)	1 (0.2%)	1 (0.2%)
Asian	38 (5.5%)	8 (1.5%)	11 (2.4%)
Black or african american	119 (17.3%)	53 (10.2%)	59 (12.9%)
White	475 (69.1%)	393 (75.3%)	214 (46.6%)
Not reported	54 (7.9%)	67 (12.8%)	174 (37.9%)

Patient information: ethnicity, gender and race.

**Table 2:** Disease features.

	TCGA-BRCA	TCGA-LUAD	TCGA-COAD
Number of patient	687	522	459
Not reported	7 (1.0%)	8 (1.5%)	11 (2.4%)
Stage X	9 (1.3%)	0 (0.0%)	0 (0.0%)
Stage I	57 (8.3%)	5 (1.0%)	75 (16.3%)
Stage IA	52 (7.6%)	134 (25.7%)	1 (0.2%)
Stage IB	4 (0.6%)	140 (26.8%)	0 (0.0%)
Stage II	5 (0.7%)	1 (0.2%)	30 (6.5%)
Stage IIA	227 (33.0%)	50 (9.6%)	137 (29.8%)
Stage IIB	155 (22.6%)	73 (14.0%)	10 (2.2%)
Stage IIC	0 (0.0%)	0 (0.0%)	1 (02%)
Stage III	1 (0.1%)	0 (0.0%)	20 (4.4%)
Stage IIIA	100 (14.6%)	74 (14.2%)	8 (1.7%)
Stage IIIB	15 (2.2%)	11 (2.1%)	60 (13.1%)
Stage IIIC	43 (6.3%)	0 (0.0%)	41 (8.9%)
Stage IV	12 (1.7%)	26 (5.0%)	46 (10.0%)
Stage IVA	0 (0.0%)	0 (0.0%)	17 (3.7%)
Stage IVB	0 (0.0%)	0 (0.0%)	2 (0.4%)

Disease information: AJCC pathologic stage.

### Mutations on cancer driver genes are detectable

We next focused our analysis on cancer driver genes, we defined the cancer driver genes as being annotated “Oncogene” or “Tumor suppressor” in the OncoKB database^42^. Selected genes are *CDH1* in TCGA-BRCA, *TP53* in TCGA-BRCA, *APC* in TCGA-COAD, *KRAS* in TCGA-COAD, *TP53* in TCGA-COAD, *EGFR* in TCGA-LUAD, *KEAP1* in TCGA-LUAD, *KRAS* in TCGA-LUAD, *PTPRD* in TCGA-LUAD, *STK11* in TCGA-LUAD and *TP53* in TCGA-LUAD. We found that mutations in *TP53* were detectable in TCGA-COAD, TCGA-LUAD, TCGA-BRCA with an AUC of 0.64 ± 0.044, 0.68 ± 0.003 and 0.79 ± 0.003 respectively. *TP53* was found detectable in the 3 datasets which suggest that *TP53* is ubiquitously detectable and could have a pan-tumoral morphological signature; however *TP53* was significantly easier to find in the TCGA-BRCA dataset. In addition, *KRAS* mutations were found modestly detectable in both TCGA-COAD and TCGA-LUAD with an AUC of 0.59 ± 0.033 and 0.57 ± 0.049 respectively. Also, mutations in the *APC* gene were hardly detectable in TCGA-COAD with an AUC of 0.55 ± 0.005, *EGFR* mutations were detectable in TCGA-LUAD with an AUC of 0.66 ± 0.049. These results are similar to those of the pan-cancer study in Noorbakhsh et al*.*^15^, thus confirming their results.

### Strategies using DL-based tests as first-line diagnostic tool can optimize the diagnosis in the patient population

**Figure 2:**
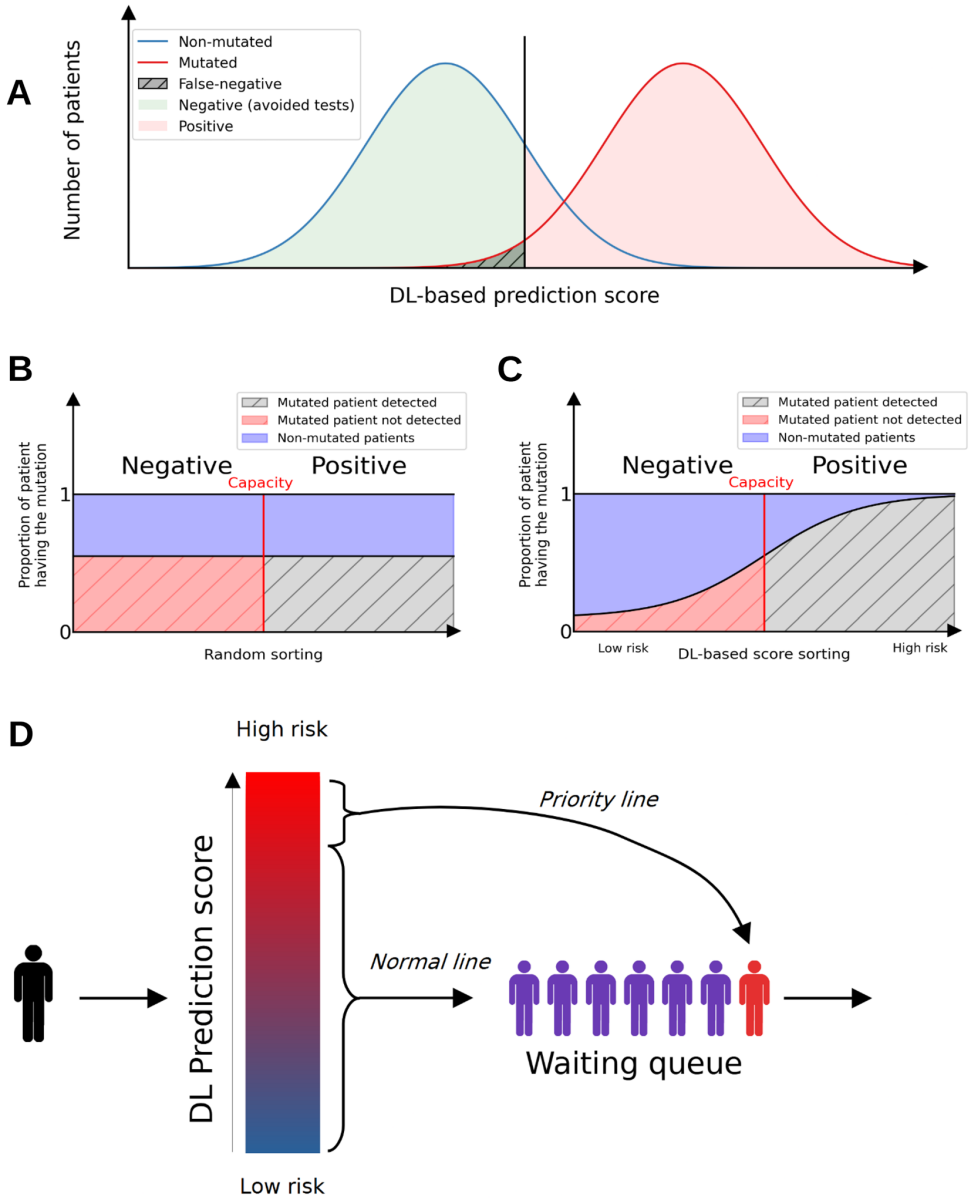
Diagnostic strategies of the deep learning triage pipeline. (**A**) Save-all strategy, red and blue gaussian curves are respectively the number of patients with and without a given mutation. The red zone corresponds to the positive patient for the DL-based diagnostic test and green zone the negative patient. The gray area is the type II error (false negative) taking the value of 5% in our different scenarii. (**B**) and (**C**) Fixed-capacity strategy respectively using the DL-based diagnostic test and using random test. The red vertical line is the limit capacity of DNA sequencing diagnostic tests, therefore all patients having a higher score than the limit capacity are diagnosed. The y-axis corresponds to the proportion of mutated patients for a given DL-based diagnostic score. The screeningDL-based strategy is expected to find more mutated patients than the random strategy using the same number of diagnostic tests. (**C**) – (**D**) Priority strategy, the blue-to-red gradient corresponds to the screening score, higher scores are in red. The top 5% or 10% patients are selected for priority diagnostic tests because they are very likely to be positive.

In the next step, we analyzed the performance of the cancer driver genes in realistic screening scenarii. We define 3 potential strategies where the DL-based test is used as a first-line diagnostic tool (Fig. 2) and show the associated performance of the algorithm as a discriminative test: (1) In the “Save-all” strategy, the DL-based test eliminates patient with low-risk of mutation from accessing sequencing tests in order to reduce costs; (2) In the “Fixed-Capacity strategy, the DL-based test conditions the access to sequencing tests when the number of these tests is limited (30% of the population can be tested, or 50%, or 70%) ; (3) in the “Prioritize” strategy, the top 5% or 10% most likely mutated patients are prioritized for an urgent sequencing test. (see Methods for further details). For each diagnostic strategy, our deep learning pipeline performance is compared to a random test.

**Figure 3:**
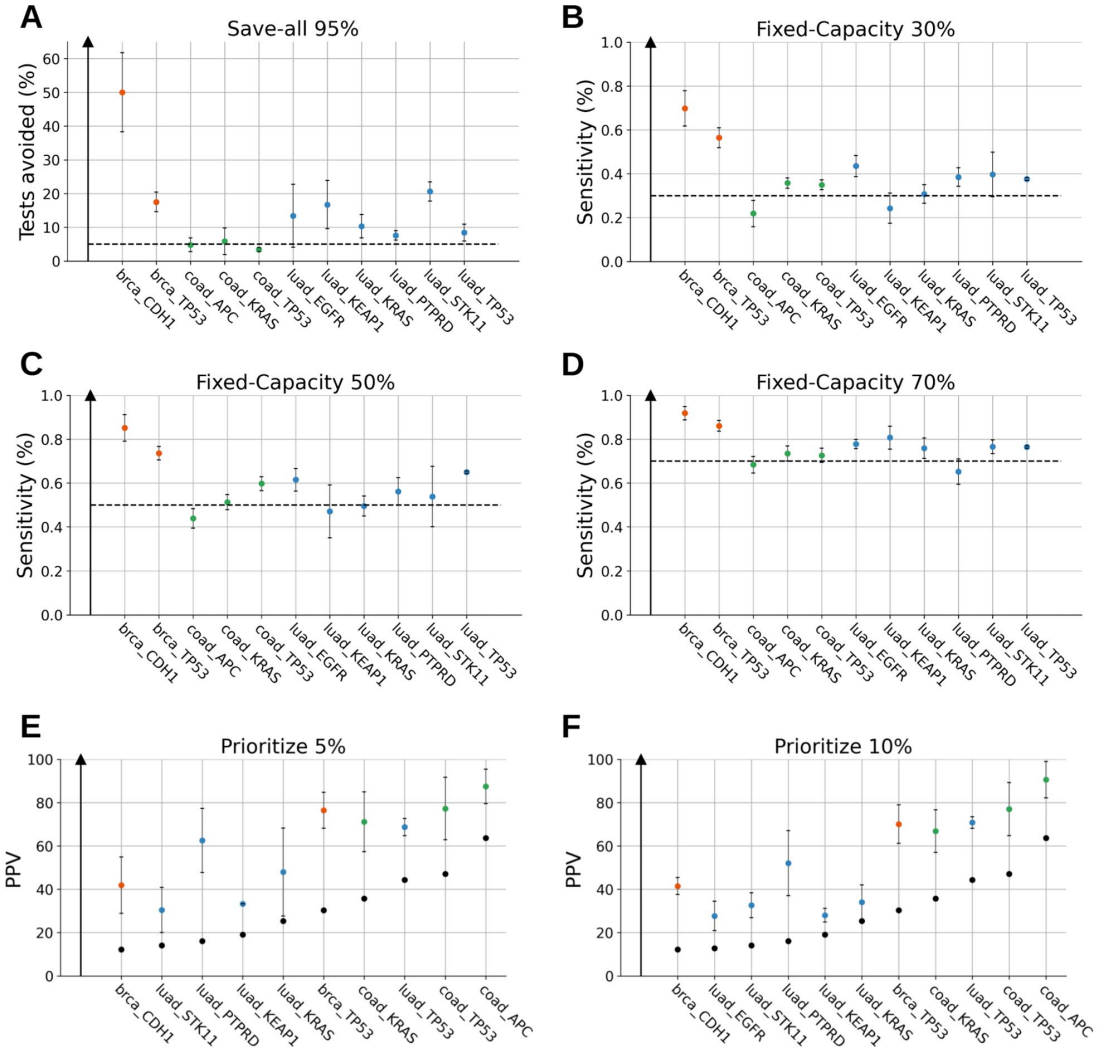
Performance of the screening pipeline for the 3 screening strategies on multiple genes. (**A**) Percentage of avoided tests at a sensitivity threshold of 5% respectively (save-all strategy). The colors correspond to the dataset of origin using the same code as in Fig. 1. The error bar shows the standard deviation of the mean. (**B**–**D**) Sensitivity for the fixed-capacity strategy with a DNA sequencing test capacity of respectively 30%, 50% and 70%. The colors correspond to the dataset of origin using the same code as in Fig. 1. The dashed horizontal line corresponds to the expected sensitivity in a random selection context. The error bar shows the standard deviation of the mean. (**E**–**F**) Positive predictive value for the priority strategy using a threshold of top 5% and top 10% selection of patients most at risk of being mutated. The colors correspond to the dataset of origin using the same code as in Fig. 1. The black dots represent the prevalence for each gene, which is the expected positive predictive value in a random context. The error bar shows the standard deviation of the mean.

In the “Save-all” strategy (Fig. 3A), proportion of avoided tests using screening range according to the gene from 3.4% to 50.0% for a 95% sensitivity. At a 95% sensitivity, sequencing could be avoided for 50.0% ± 11.7% of the population for the *CDH1* gene in TCGA-BRCA, for 20.6% ± 2.8% of the population for *STK11* gene in TCGA-LUAD and for 17.4% ± 2.9% of the population for *TP53* gene in TCGA-BRCA. Interestingly, the performance of the strategy is negatively correlated with the prevalence (pearson R = -0.575, p-value = 0.064), therefore genes being rarely mutated are prime targets for the “Save-all” strategy.

In the “Fixed-Capacity” strategy (Fig. 3B-D), the global sensitivity (sensitivity of the 2-step DL test + sequencing test diagnostic) varied according to the gene from 21.8% to 69.8% at a 30% capacity, from 43.8% to 85.1% at a 50% capacity, and from 65.2% to 91.9% at a 70% capacity. These values have to be compared with the value of a random test equaling the capacity (e.g. 30% for a 30% capacity). For example, with the *APC* gene from the TCGA-COAD dataset, the strategy is performing lower than the theoretical random test; this effect is probably due to stochastic variability on an uninformative DL test. At a 30% capacity, CDH1 gene in TCGA-BRCA showed 69.8% ± 8.1% sensitivity, TP53 gene in TCGA-BRCA showed 56.5% ± 4.5% sensitivity and EGFR in TCGA-LUAD gene showed 43.5% ± % sensitivity. At a 50% capacity, CDH1 gene in TCGA-BRCA showed 85.1% ± 6.0% sensitivity, TP53 gene in TCGA-BRCA showed 73.5% ± 3.0% sensitivity and TP53 gene in TCGA-LUAD showed 65.0% ± 0.5% sensitivity. At a 70% capacity, CDH1 gene in TCGA-BRCA showed 91.9% ± 3.0% sensitivity, TP53 gene in TCGA-BRCA showed 86.1% ± 2.4% sensitivity and KEAP1 gene in TCGA-LUAD showed 80.7% ± 5.2% sensitivity. In this setting, the DL-test does not clearly show up as a relevant clinical strategy. However, training with more data and a more sophisticated pipeline could help improve the performance of this DL-test and optimize sequencing in the case of a limited availability of DNA sequencing test.

In the “Prioritize” strategy (Fig. 3EF), the positive predictive value (PPV) of the DL method varied from 30.4% to 87.5% for the top 5% of patients most at risk of being mutated for a specific gene. To better estimate the impact of the strategy, we also calculated the relative risk (RR) of having a given mutation through the normalization with the gene mutation prevalence (i.e. the ratio of the PPV over the prevalence, which corresponds to a relative increase in PPV) in order to have a fair comparison with the theoretical random test. For the top 5% of patients most at risk, the RR varied according to the gene from 1.375 ± 0.12 to 3.886 ± 0.91. The CDH1 gene in TCGA-BRCA showed a 41.9% ± 13.0% PPV and a 3.43 ± 1.06 RR, the PTPRD gene in TCGA-LUAD showed a 62.5% ± 14.7% PPV and a 3.89 ± 0.91 RR and the APC gene in TCGA-COAD showed a 87.5% ± 7.9% PPV and a 1.38 ± 0.12 RR. For the top 10% of patients most at risk of being mutated, the PPV varied from 27.7% to 90.6% and the RR varied from 1.345 ± 0.31 to 3.390 ± 0.31. The CDH1 gene in TCGA-BRCA showed a 41.5% ± 3.9% PPV and a 3.39 ± 0.31 RR, the PTPRD gene in TCGA-LUAD showed a 52.1% ± 15.0% PPV and a 3.24 ± 0.93 RR and finally the APC gene in TCGA-COAD showed a 90.6% ± 8.4% PPV and a 1.42 ± 0.13 RR. In this proof of concept article, these results show the relevance of this DL test to prioritize patients with highest risk of being mutated for a given gene. Additionally, we can notice that the RR is correlated with the prevalence (pearson R = -0.68, p-value = 0.019 for top 5%, pearson R = -0.60, p-value = 0.047 for top 10%). However, this does not mean that the tests are intrinsically better for low prevalence genes. In order to question this relation, one should compare prevalence with odds ratios which are independent of the prevalence. In our case, when odds ratios are calculated, we do not find any correlation (pearson R = -0.122, p-value = 0.72 for top 5%, pearson R = 0.25, p-value = 0.45 for top 10%), thus showing no significant link between prevalence and intrinsic test performance (i.e. test evidence).

### The fixed-capacity strategy is efficient to reduce population inequalities

**Figure 4:**
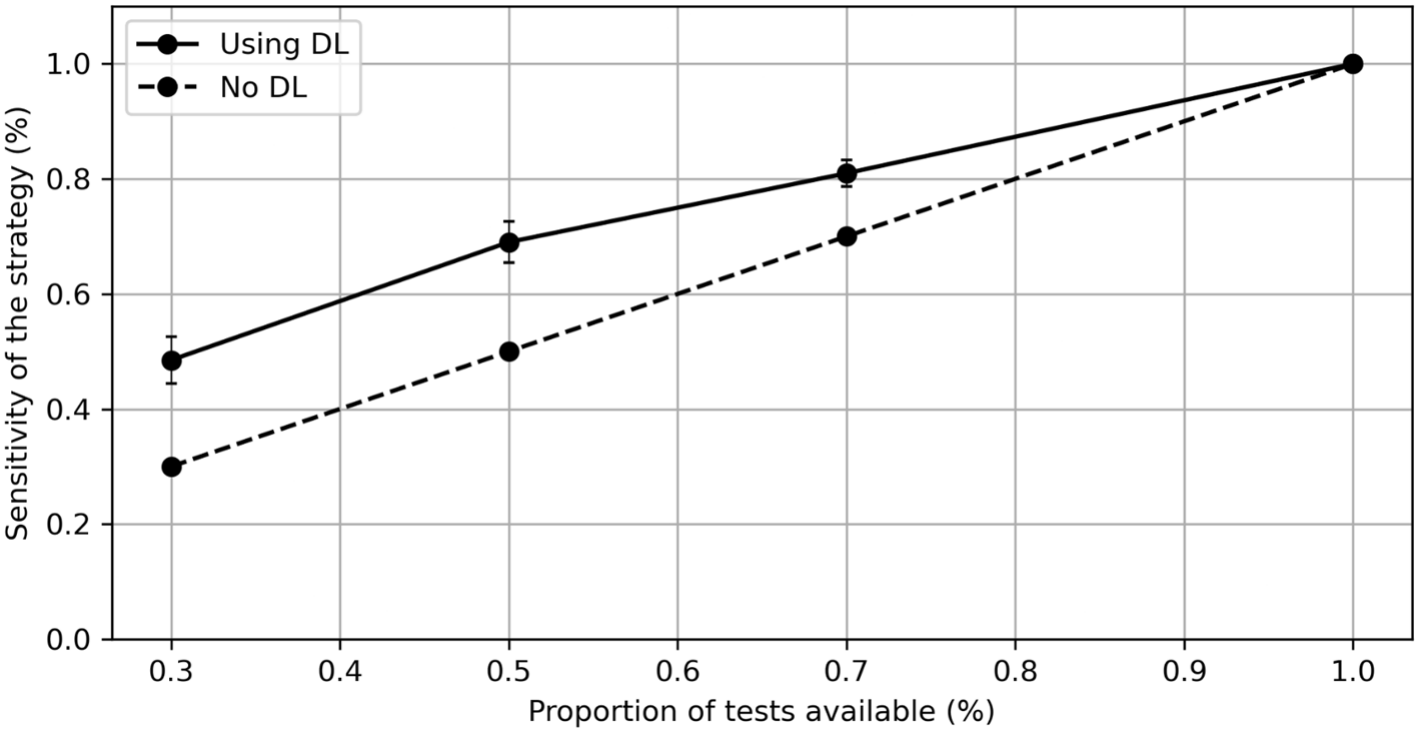
Inequality reductions with the use of Deep Learning (DL). Plot of the sensitivity of the fixed capacity strategy in a population according to the proportion of sequencing tests available, and with or without the use of Deep Learning as a first-line diagnostic tool. The 5 cancer driver genes with highest sensitivity were gathered and averaged in order to create a realistic multi-gene application of the fixed-capacity strategy.

We next analyzed the effect of using our deep learning pipeline in a context of unequal access to DNA sequencing, as we defined it earlier as a “fixed-capacity” scenario. As previously mentioned, we defined the sensitivity of the entire diagnostic pipeline as our metric of interest. We compared the difference of sensitivity for the population having 30%, 50% and 70% of available tests like in the previous configurations. We also added a theoretical population having 100% tests available. To have a more accurate estimation, we only kept the top 5 genes with the highest performances in the “fixed-capacity” strategy, namely CDH1 in TCGA-BRCA, TP53 in TCGA-LUAD, TP53 in TCGA-COAD, EGFR in TCGA-LUAD and TP53 in TCGA-BRCA. We show that the sensitivity gap is reduced between unequal populations (Fig. 4). For a 100% capacity there is obviously no sensitivity increase using DL methods, while for a 30% capacity, there is an absolute increase of 19 percentage points, thus reducing the gap between the low-access population (30%) and the high-access population (100%) by 27%, and from 70 percentage points to 51 percentage points (Fig. 4). One can expect that the effect will increase with the performance of the DL method.

## Discussion

Cancer is a very heterogeneous disease where no treatment is ubiquitously efficient. Targeted therapies have emerged in response to this heterogeneity, as an example, the FDA approved 20 more targeted therapies between April 2019 and April 2021^43^. However, these treatments require acquiring more and more molecular information about the tumour.. Acquiring such information is still difficult because of the infrastructure needed, its price and public reimbursement policies^43^, therefore making their democratization difficult.

In this paper, we propose plausible diagnostic strategies based on new deep learning methods, and a way to evaluate their impact on clinical practice using standard sensitivity of PPV values. These deep learning methods are faster, more cost-effective, scalable and easily integrable in the current diagnostic workflow. We show that predicting mutational status from WSI can optimize the allocation of diagnostic test resources in 3 credible scenarii for various cancer driver genes. Moreover, we demonstrated that the fixed-capacity is efficient to compensate for inequalities between populations. Thus, DL methods may help reduce the inequality gap between low or middle income countries and high income countries, but even within high-income countries^43^ as reducing inequalities in the access to diagnosis and treatment is a key priority of the European Union ^44^. These DL methods could also ultimately be a tool for public administrations to control their budgets and optimize their health policies.

Our results suggest that using a Deep Learning pipeline as a first-line diagnostic is particularly relevant for genes having low prevalence in the Prioritize and the Save-all strategies. However, having a low prevalence also implies that finding a mutated patient is more difficult and that it might be harder to reach a satisfying level of performance. The Save-all strategy with low prevalence mutations is analogous to a high sensitivity strategy on rare disease, which is generally known as a screening strategy and is one of the most obvious applications for such a DL pipeline because of the massive cost reduction for finding a positive patient by reducing the number of DNA sequencing. However, a medico-economic analysis should be made to precisely describe the benefits of a deep-learning based genomic screening for cancer patients. Such an analysis should integrate the DNA sequencing cost and availability and its clinical benefits for a considered targeted therapy (e.g. life years gained).

This study is a preliminary study and has clinical limitations. First the lack of a true external dataset to validate the results on non-TCGA data. Results are dependent on the prevalence of the mutation, which is here approximated by the prevalence in the datasets but might not be the same in real application. Finally, we used a standard baseline DL method on a limited dataset as a proof of concept, but it does not show the state-of-the-art of the technology. A similar analysis with state-of-the-art methods and a larger dataset should be made to better evaluate the potential of the technology and refine the potential of the strategies. Clinical implementation would require better and clearer performances validated at least in an external dataset in order to reach regulatory compliance, or in a prospective trial in order to have robust conclusions about the true clinical value as stated in van der Laak et al*.*^45^.

## Data Availability

The data analyzed in this study were obtained from The Cancer Genome Atlas (TCGA), which is publicly available through the Genomic Data Commons (GDC) Data Portal (https://portal.gdc.cancer.gov/). The GDC Data Portal provides access to a variety of TCGA data types, including genomic, transcriptomic, proteomic, and clinical data from thousands of tumor and normal tissue samples across multiple cancer types. Data can be downloaded through the GDC Data Transfer Tool or accessed through the GDC Data Portal API. Detailed information on the available data and instructions for accessing and downloading the data are provided on the GDC Data Portal website. All data used in this study were used in accordance with the GDC Data Use Policy.
